# Gustav Oppenheim (1882–1937) and the Discovery of Cerebral Amyloid Angiopathy

**DOI:** 10.1177/10738584241251828

**Published:** 2024-05-14

**Authors:** Anthony Maurice Ness, Judd Aiken

**Affiliations:** 1 Department of Biological Sciences, Centre for Prions and Protein Folding Diseases, University of Alberta, Edmonton, Canada; 2 Department of Agricultural, Food & Nutritional Science, Centre for Prions and Protein Folding Diseases, University of Alberta, Edmonton, Canada

**Keywords:** cerebral amyloid angiopathy, amyloid β, Alzheimer disease, neurology, history of medicine, neuroscience

## Abstract

The discovery of cerebral amyloid angiopathy (CAA) is frequently attributed to Dr. Gustav Oppenheim—a man who has been largely passed over in history. Oppenheim’s clinical and neuropathologic research covered a variety of disorders, but his name is best known for his work on senile dementia and CAA. Although Oppenheim was in fact not the first to discover CAA, his neuropathologic observations and inferences on neurodegenerative disease proved to be remarkably faithful to our modern understanding of neurodegenerative diseases. As a neurologist, he served in the First World War and was later subjected to religious persecutions in the leadup to the Holocaust but was not fortunate enough to emigrate before his death. The life, social impact, and previously overlooked contributions to science and medicine by Oppenheim are detailed.

## Introduction

Cerebral amyloid angiopathy (CAA) refers to the pathologic deposition of extracellular amyloid β peptide within cerebral capillaries or larger vessels. Development of CAA neuropathology is typically age related but has genetic risk factors (Banerjee and others 2023; Thal and others 2008). The amyloid β peptide also constitutes Alzheimer disease–associated amyloid plaque neuropathology, although CAA can co-occur as a pathologic feature of Alzheimer disease or occur independently (Greenberg and others 2020; Thal and others 2008). Cerebrovascular dysfunction caused by CAA is linked to cerebral hemorrhages of varying degrees and cognitive impairment (Banerjee and others 2023; Greenberg and others 2020). Iatrogenic transmission of CAA has been recognized (Banerjee and others 2023; Greenberg and others 2020). The risk of intracranial hemorrhage associated with CAA may affect patient treatment considerations, notably for prescribing of anticoagulants and statins (Banerjee and others 2023).

**Figure 1.**
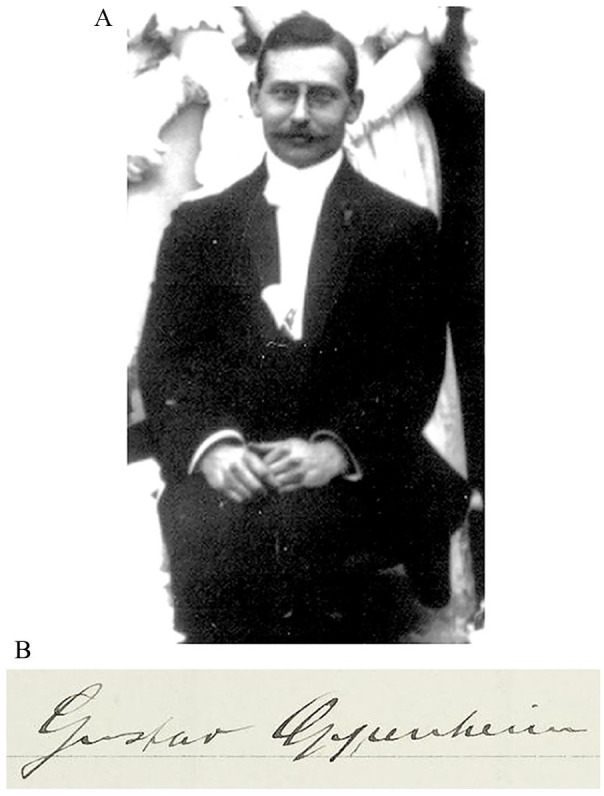
(A) Gustav Oppenheim at the wedding of Leopold Wallersteiner and Elsa Bergmann in Laupheim, Württemberg, on November 19, 1905. Image courtesy of the Gesellschaft für Geschichte und Gedenken Laupheim e.V. (B) Oppenheim’s signature upon his entry into the Heidelburg University medical program in the summer semester of 1903.

The discovery of CAA is often incorrectly referred to a 1909 article by Gustav Oppenheim, but little is known of the man himself, the context of his pioneering research, or his later life. Oppenheim was born on February 16, 1882, in Frankfurt am Main (Frankfurt/M), the German Empire, to Mortiz and Hilda Oppenheim, née Simon—a Jewish merchant family involved in soap, candle, and perfume making (Krug 1883; Oppenheim 1950–1969). After initially studying in Berlin, he entered the medical program at Heidelberg University (Ruprecht-Karls-Universität Heidelberg) in 1903, successfully defending his dissertation on vascular tumor metastasis (Oppenheim 1905) and passing the medical state examination in 1905 (Figure 1; AVFM 1908).

**Figure 2.**
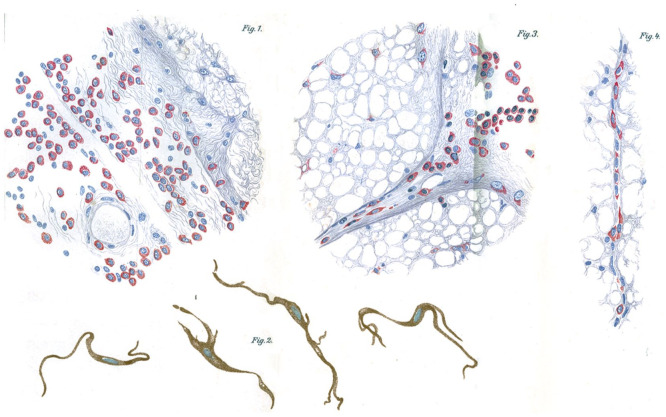
Plasma cell infiltration of the spinal cord and pigmented cells found in the spinal pia in cases of neurosyphilis described by Oppenheim in 1908.

The newly minted physician accepted a position as a clinical assistant for neuropathologist and psychiatrist Professor Alfred Hoche (1865–1943) from 1905 to 1908 at the University of Freiburg Psychiatrische und Nervenklinik (Psychiatric and Mental Hospital). Oppenheim published clinical and neuropathologic observations on neurologic disorders, including multiple sclerosis and “progressive paralyse”—general paresis caused by neurosyphilis (Figure 2; Oppenheim 1908a, 1908b). During his time in Freiburg, Oppenheim worked alongside the neuropathologist Walther Spielmeyer (1879–1935), who was also an assistant under Dr. Hoche from 1902 to 1912 (Spatz 1935). Together the two made neuropathologic observations of multiple sclerosis, with Oppenheim developing a histologic method for visualizing the pathology of the disease (Spielmeyer 1910, 1924). Spielmeyer would later be appointed by Emil Kraepelin (1856–1926) in 1912 to succeed Alois Alzheimer (1864–1915) as the head of the Anatomischen Laboratiums der Psychiatrischen Klinik (Laboratory for Neuropathology) in Munich when Alzheimer fell ill and resigned from his post (Hippius and Müller 2008; Spatz 1935).

In 1920 the former supervisor of Oppenheim, Dr. Alfred Hoche, released the infamous ‘Die Freigabe der Vernichtung lebensunwerten Lebens: Ihr Maß und ihre Form’ (The extent and form of the legalization of the destruction of lives that are no longer worthy), coauthored by the retired professor of law Karl Binding (1841–1920; Wolfensberger 1993). The book advocated for forced euthanasia of those who were brain damaged or mentally disabled. The writing would be used by the Nazis to justify the Aktion T4 involuntary euthanasia murder program. In contrast, records suggest that Oppenheim was opposed to eugenics. In a 1910 Frankfurt/M Medical Association meeting, Oppenheim argued against sterilizing mentally ill women—instead arguing that not all the women were mentally ill and that “the prognosis of the incurability of mental disorders is uncertain” (AVFM 1910).

Following the end of his work in Freiberg as a clinical assistant in Hoche’s laboratory in 1908, Oppenheim returned to his birthplace of Frankfurt/M to begin work in 1909 as a private researcher and neurologist, where he continued his clinical research on multiple sclerosis histopathology and neurosyphilis (Oppenheim 1910; von Juliusberg and Oppenheim 1911). In 1909, he published his observations on senile amyloid plaques, including the discovery of CAA, in the German academic journal Neurologisches Centralblatt (Oppenheim 1909).

## Oskar Fischer, Gustav Oppenheim, and the Discovery of CAA

Alois Alzheimer’s 1906 conference case report of the Frankfurt/M patient August Deter described what would become known as Alzheimer disease (Alzheimer 1907; Hippius and Neundörfer 2003). The published notes of the lecture were short, and the only descriptions of the cerebral vasculature made note of arteriosclerotic change in the larger cerebral vessels; otherwise, there was a lack of vessel involvement. Oskar Fischer (1876–1942) at the German University of Prague published a more descriptive 1907 neuropathologic paper on 16 patients with senile dementia but did not identify cerebrovascular involvement (Fischer 1907; Goedert 2009). Fischer observed plaques in 12 of 16 patients with senile dementia but did not report plaques in 10 control brains, 10 psychosis brains, or 45 neurosyphilis brains (Goedert 2009). Fischer linked the presence of plaques in the brain to presbyophrenia—a now outdated subclass of dementia coined in 1863 by Karl Ludwig Kahlbaum (1822–1899) characterized by memory impairment, disorientation, confabulation, euphoria, and agitation (Berrios 1994; Kahlbaum 1863).

Oppenheim’s 1909 article is the first scientific publication describing CAA; however, Oppenheim was not the first to report the existence of CAA (Oppenheim 1909). Oppenheim acknowledges that he replicated cerebrovascular observations presented by Oskar Fischer at the Jahresversammlung des Deutschen Vereins für Psychiatrie (Annual Meeting of the German Association for Psychiatry) in Berlin on April 24, 1908. Fischer’s talk titled ‘Zur Histopathologie der Presbyophrenie’ (On the histopathology of presbyophrenia) outlined the examination of 37 cases of senile dementia (Fischer 1908). Fischer reported finding plaques in all 28 patients diagnosed with presbyophrenia using Bielschowsky silver stains. As with Fischer’s 1907 publication, no plaques were observed in numerous control brains from 30 patients with other neurologic diseases and 20 patients without dementia. Fischer stated that he recognized a very close association between the amyloid material and the capillaries and larger vessels. No inflammatory response was noted. Similarly appearing neurovascular pathology had been reported in elderly patients with stroke by Jean-Baptiste Ferrand (1873–1957) in his 1902 thesis using Marchi osmic acid stains (Ferrand 1902). The stained granular material inside the cerebral vessels observed by Ferrand were instead components of degenerated myelin sheaths (Strich 1968).

Fischer’s interpretation of the results was that the drusen were caused by *streptotricheen*—referring to filamentous actinomycetes bacteria (thought by many at the time to be a fungus) that causes dermatophilosis (Hyslop 1979; Langer 1904; Peemöller 1922; Stackebrandt and Schumann 2006). Fischer claimed that these plaque-forming structures were Gram negative and not acid fast. Attempts to culture the speculated bacteria failed. During the discussion period, a “Herr Vogt” from Frankfurt—possibly Dr. Heinrich Vogt (1875–1957)—stated that he had personally made similar observations (no published results) and contested Fischer’s assumption of a fungal origin of drusen. Fischer’s results would not be published until 1910 (Fischer 1910).

Oppenheim’s 1909 ‘Über “drusige Nekrosen” in der Großhirnrinde’ (About necrotic plaques in the cerebral cortex) compared his neuropathologic notes on senile dementia with those of Fischer’s 1907 paper and 1908 oral presentation (Fischer 1907, 1908; Oppenheim 1909). At the time, ‘drusen’ or ‘drusige nekrosen’ referred to what are now known as amyloid plaque pathology. As aptly explained by Michel Goedert, the ‘drusen’ translated to geode—a comparison of fibrillar amyloid plaques to the hollow spheroid geodes with internally crystalline structures (Goedert 2009). Oppenheim examined 14 autopsied brains of patients with senile dementia using Nissl and Bielschowsky staining methods. Oppenheim also examined 2 human aged control brains without dementia received from Professor August Knoblauch (1863–1919), director of the Frankfurt/M Urban Infirmary. Plaques were identified in 6 of 14 senile dementia brains, including 1 with asymmetrical atrophy of the left temporal lobe associated with plaques. The structural findings of the plaques and neurofibrillary tangles were nearly identical to those of Fischer; however, Oppenheim’s insightful article did not restrain from pointed remarks that directly challenged Fischer with differing observations that are more faithful to our current knowledge of the aging brain and senile dementia. Unlike Fischer, Oppenheim identified a comparably extensive abundance of plaques in one of his 2 aged nondementia control brains (from a 70-year-old man). Oppenheim’s observation of extensive proliferative gliosis in the vicinity of the plaques also conflicted with Fischer’s observations. Oppenheim concluded that only 3 of his 6 presbyophrenic cases accumulated amyloid plaques, in contrast to Fischer’s proclaimed absolute link between drusen and presbyophrenia.

Oppenheim’s insightful clinicopathologic observations of senile dementia and his more astute interpretations attest to the quality and objectivity of his work as a neurologist. Regarding the cerebrovasculature, Oppenheim noted that amyloid staining appeared more commonly in and surrounding capillaries with a “glass-hyaline” texture, but he alluded to larger vessels being affected as reported by Fischer. This was not only the first published description of CAA but also that of the dilated perivascular space caused by CAA and the first use of “hyaline” to describe the perivascular degeneration. Similar to Vogt, Oppenheim contested Fischer’s assumption that the plaques were caused by streptotriche-like microorganisms. Oppenheim concluded his article with a conjecture that the plaques were instead caused by a “lifeless substance” that could be chemically studied.

**Figure 3.**
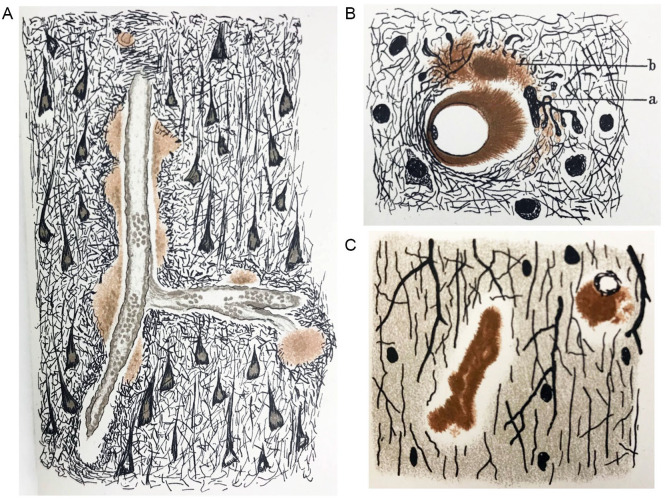
The first known drawings of cerebral amyloid angiopathy by Fischer in 1910: (A) larger cerebral vessels (100× enlargement), (B) capillaries with dilated perivascular space (500× enlargement), and (C) capillaries (600× enlargement).

Following the criticism of Fischer’s streptotricheen theory in the 1908 Berlin meeting, Fischer withheld his neuropathologic work on the subject until 1910, when he published a colossal 100-page paper (not including 51 additional figures) detailing his neuropathologic observations of senile dementia, including those outlined in his 1907 paper and 1908 speech (Fischer 1910). Fischer reiterated his theory of a near-absolute link between plaques and presbyophrenia and coined a new but short-lived phrase for the plaque pathology—*Sphaerotrichia cerebri multiplex*. Fischer re-emphasized the similarities between plaques and the filamentous/spheroid bacteria but stopped short of stating that the plaques were caused by a fungus or bacteria (Fischer 1910). Fisher contradicted findings by his contemporaries, including those of Oppenheim and Gaetano Perusini (1879–1915) from the Alzheimer research group. Fischer acknowledged Oppenheim’s 1909 observations of plaques in nondementia controls but was skeptical of his conclusion that plaques were not absolutely linked to presbyophrenia. Regardless of his erroneous interpretations, Fischer’s paper would become the most intensive neuropathologic study of neuritic plaques in senile dementia for the time and would include the first known drawings and photomicrographs of CAA (Figure 3).

## First World War Service and Later Antisemitic Persecution

**Figure 4.**
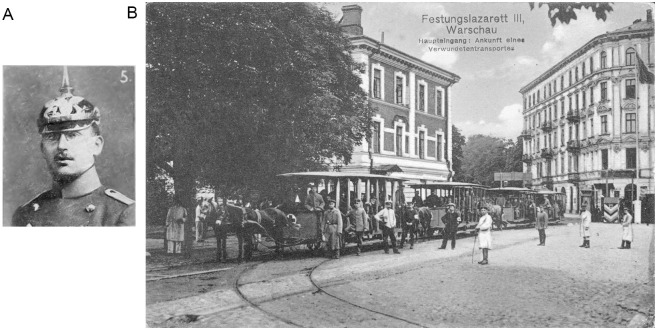
(A) Oppenheim as an Oberarzt medical officer during the First World War in 1915 when he was awarded the Iron Cross. The ornamental front plate on the spiked helmet (Pickelhaube) then worn by German soldiers denoted the regiment’s province or state (Baden in this case). (B) Wounded arriving at the Military Hospital III in Warsaw (renamed from Ujazdowski Military Hospital), 1917, the complex where Oppenheim led a psychiatric ward. Image from the Biblioteka Narodowa.

**Figure 5.**
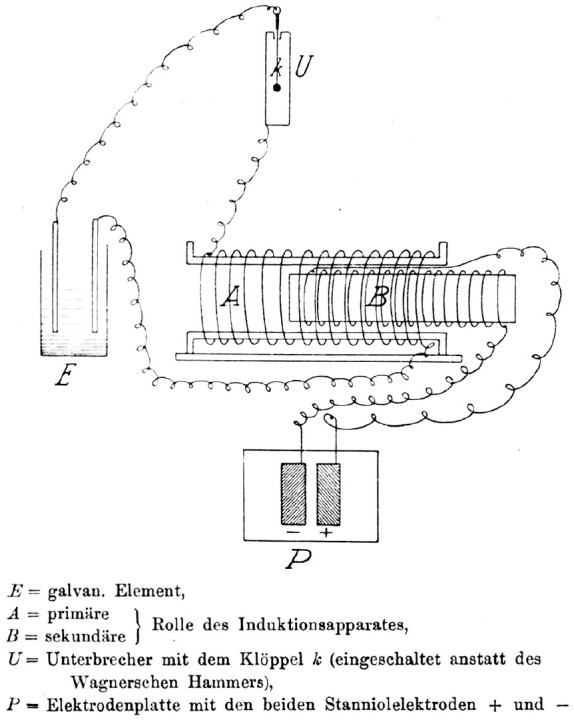
Circuit diagram of an electrical device used by Oppenheim to treat psychiatric patients with tremors during the First World War. Aluminium electrodes (P) were fixed to the middle of the patient’s back. An induction apparatus (A/B) held or attached to the patient caused painful shocks only when the apparatus was moved.

Oppenheim settled in Frankfurt/M and married Alice (Liesel) Reis (b. 1891) on January 16, 1912 (Dr. med. 1912), a marriage that would last until his death. Oppenheim was a member of a private neurology polyclinic, the Frankfurter Poliklinik für Nervenkrankheit, from approximately 1910 until the start of the First World War (Adressbuch 1910). Oppenheim served with the German armed forces in the field as an Oberarzt (senior physician) of the Reserve attached to the Reserve Eisenbahn-Bau Kompanie Nr. 16 (the 16th Reserve Railway Construction Company) from the start of the war in August 1914 until July 1916 (Kallmorgen 1936). During his service, he earned the Iron Cross in 1915 (Figure 4A) and the Baden War Merit Cross (Kriegsverdienstkreuze) in 1916 (Frankfurter 1915; Oppenheim 1916–1918). Oppenheim was then promoted to the rank of Stabsarzt (staff surgeon) and transferred to head a military psychiatric ward in the Festungslazarett III, Warschau (Military Hospital III in Warsaw, formerly the Ujazdowski Military Hospital; Figure 4B), until the end of the war (Kallmorgen 1936). At his new post in the military medical services mental health department, Oppenheim published a paper on an invention intended for the treatment of German soldiers with combat stress reaction–related tremors using electrotherapy (Figure 5; Lerner 2003; Linden and Jones 2013; Oppenheim 1917). The device was designed to provide a painful electric shock whenever the tremors set in—providing a painful punishment conditioning response with the intent for soldiers to learn to control their tremors.

After the war, Oppenheim’s clinical research continued to center on electrotherapy and included being granted two patents on electrodiagnostic and electrotherapy devices (Dr. med. 1938; Oppenheim 1928, 1933a, 1933b). Oppenheim resumed his practice at the same Frankfurt/M polyclinic until 1922 (Adressbuch 1923) when he set himself on a new calling. In 1922 Oppenheim was elected (Dr. med. 1938) the chair of the Aerzte-Verbandes für freie Arztwahl (Doctors Association for the Free Choice of Physicians), a professional medical association with a very politically charged name. The medical association was functionally unusual for its time. The association managed funds raised from wealthy community members to run a clinic in Frankfurt/M that provided free medical treatment and medications for the poor, while providing financial assistance to doctors in need (Hanauer 1923). The charitable association was lauded in 1923 for its progressive and benevolent organization (Hanauer 1923).

Oppenheim continued his research and clinical neurology work in Frankfurt/M, Germany, until 1933 when he, with thousands of other Jewish physicians and academics across Germany, was swept up by the anti-Semitic policies and public incitement of the Nazi party (Kater 1989b). On March 28, 1933, the Frankfurt/M city administration ordered the dismissal of Jewish civil servants (Aly and others 2019). After 11 years of being chair of the Doctors Association for the Free Choice of Physicians, Oppenheim was removed from his post for being Jewish in March 1933. He also began losing patients in his private practice (Oppenheim 1950–1969). The fate of the medical association and its charitable clinic after 1933 is unclear, but the year coincides with the Gleichshaltung (consolidation into totalitarian control) of medical professional organizations and sickness funds by the Nazi party (Busse and Blümel 2014; Kater 1989a).

The Law for the Restoration of the Professional Civil Service in April 1933 began the systematic dismissal of Jewish faculty and staff from German universities (Aly and others 2019; Grüttner 2022) and resulted in Jewish physicians losing many patients when the state statutory health insurance system would no longer cover patients treated by Jewish doctors (Aly and others 2019; Busse and Blümel 2014; Kater 1989b). Exceptions granted for First World War front-line service or familial losses were removed in the September 1935 Nuremberg Race Laws (Kater 1989b). Revenue dried up when contracts between Jews and private insurance funds were abrogated in 1933, all while the German public was encouraged and sometimes coerced to avoid Jewish physicians (Kater 1989b).

**Figure 6.**
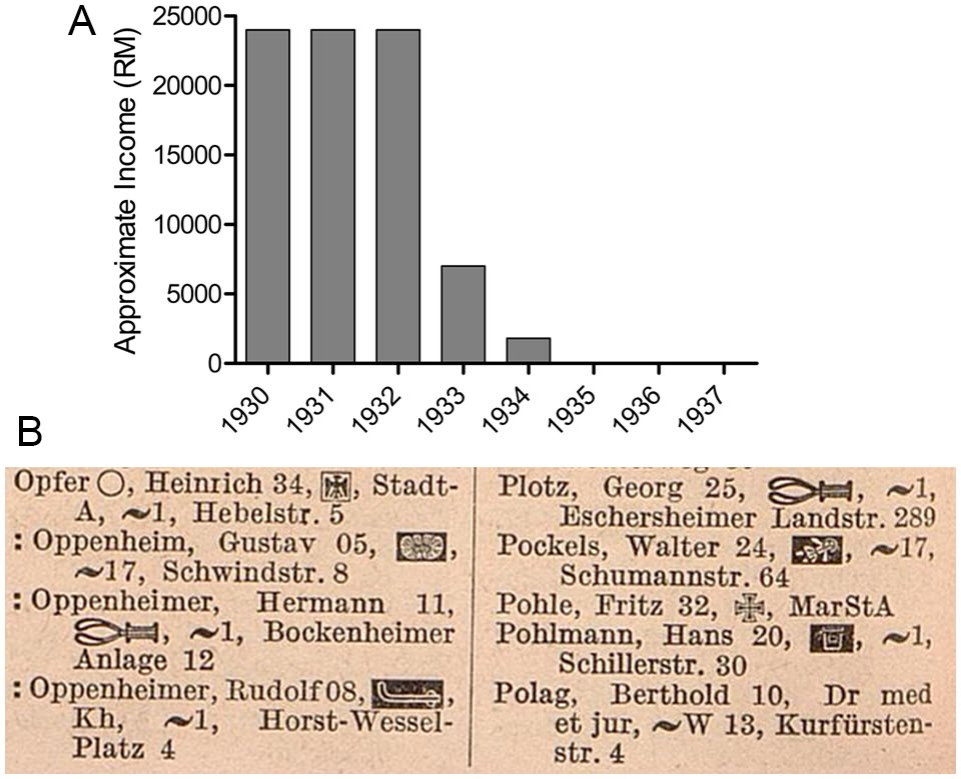
(A) Oppenheim’s estimated income in the 1930s as later reported by his widow. (B) Oppenheim’s name in the 1937 registry of German physicians. Colons were placed before the names of Jewish physicians in the 1937 edition as a means of stigmatization. The symbols after the names indicate specializations or military postings. The symbol next to Oppenheim’s name indicates a specialization in neurologic diseases, but a second symbol for a specialty in psychiatry that had been present since the 1910 registry was absent from the 1937 edition for unknown reasons.

Oppenheim’s widow and colleagues claimed that he was briefly jailed in 1933, although accounts differ on whether he detained in March or May (Oppenheim 1950–1969). According to his widow, Oppenheim and 27 other prominent Jewish citizens, businessmen, lawyers, and physicians were arrested in Frankfurt/M and taken to the Old Town prison in wagons, where Oppenheim was detained for a few days (Oppenheim 1950–1969). Although a specific event cannot be independently identified, the description is consistent with the humiliating Prangermärsche (pillory marches) often targeting Jews across Germany that year (Beck 2022). Following his imprisonment, Oppenheim suffered a severe mental breakdown and depression and underwent personality changes for which he spent time at a Baden spa, although his nerves never fully recovered (Oppenheim 1945–1958, 1950–1969). Oppenheim’s widow and a former medical association colleague later estimated that his income had plummeted from approximately 24,000 Reichsmarks (RM) in 1932 (approximately US $5700 at the time) from his chairship and private practice to 7000 RM in 1933 (Oppenheim 1950–1969). His income further declined to 1800 RM in 1934 and then to almost none thereafter (Figure 6A; Oppenheim 1950–1969). As with other Jewish physicians, Oppenheim’s name was marked in the 1937 German physician’s directory (Figure 6B; Lautsch and Dornedden 1937).

The loss of patients and exclusion from opportunities at research institutions likely contributed to Oppenheim shifting into pseudoscience—likely out of desperation. The quality of Oppenheim’s research suffered a precipitous fall. A patent was granted to Oppenheim in 1935 for a simplistic and rudimentary device for “Franklinization”—a quackery-based therapy wherein charging a person with static electricity was thought to provide therapeutic benefits (Oppenheim 1935). The invention consisted of superimposing sheets of different materials, such as rubber and silk, followed by rolling the materials to generate static electricity with the intention of transferring the charge to a human. In 1936 Oppenheim traveled to Amsterdam to give a showman-styled lecture regarding “electric fields on the human body and their significance for parapsychological phenomena,” with separate laboratory demonstrations of static electricity (De 1936). The field of parapsychology—the study of human-controlled paranormal and psychic phenomena—had reemerged in the early 20th century with academic proponents including the aforementioned neurologist Fischer, Nobel laureate and immunologist Charles Richet (1850–1935), and physicist Pascual Jordan (1902–1980; Goedert 2009; Pascual 1951; Wolf 1993).

A colleague attested that Oppenheim developed symptoms of an unspecified “blood disease” in 1936 (Oppenheim 1950–1969). In late November 1937, Oppenheim was hospitalized at a Frankfurt/M Jewish hospital, where he died 10 days later on December 5, 1937, at age 55 (Oppenheim 1945–1958, 1950–1969). Oppenheim’s widow, Alice, fled Germany with her two children in early 1939 (G 1975; Oppenheim 1950–1969). During emigration from Germany, Alice arranged for the transport of her belongings and furnishings; however, they never arrived and are understood to have been intercepted, confiscated, and auctioned off by the Gestapo (Oppenheim 1950–1969). More devastating, Alice was separated from her son during emigration. Alice ultimately settled in London, England; she remained a widow until her death in 1975 (G 1975).

A reconstruction of the life and work of Oppenheim reveals a talented and objectively thinking clinical researcher. As a neurologist, Oppenheim studied and treated a range of diseases. A retrospective look at Oppenheim’s research on neurodegenerative diseases confirms that he was a pioneering neurologist. His observations and interpretations of CAA and amyloid β neuropathology remain faithful to modern medical knowledge despite conflicting opinions of colleagues at the time. The drive for conducting clinical research continued into the Great War and later during the interwar period. In his later life, Oppenheim dedicated himself to clinical practice and serving the local Frankfurt/M community before having his life upended by the Nazi regime.
